# Dynamic Evolution and Convergence of the Coupled and Coordinated Development of Urban–Rural Basic Education in China

**DOI:** 10.3390/e27101021

**Published:** 2025-09-28

**Authors:** Fangyu Ju, Qijin Li, Zhiyong Chen

**Affiliations:** School of Mathematics and Statistics, Fujian Normal University, Fuzhou 350117, China; jufangyu@fjnu.edu.cn (F.J.); chenzy@fjnu.edu.cn (Z.C.)

**Keywords:** urban–rural, basic education, coupled and coordinated development, dynamic evolution, convergence

## Abstract

Understanding the coupled and coordinated development of China’s urban and rural basic education systems is crucial for fostering their interaction and synergistic growth. Using China’s provincial panel data from 2011 to 2023, this study measures the coupled and coordinated development level of urban–rural basic education (CCD-URBE) via the entropy weight method, G1-method and coupling coordination degree model. On this basis, the Dagum Gini coefficient decomposition method, traditional and spatial Markov chain models, as well as convergence test models are employed for empirical research. The results show that: (1) During the study period, the CCD-URBE across the nation and the four major regions improves significantly. Both intra-regional and inter-regional disparities show a consistent downward trend. Inter-regional disparities are the main source of the overall disparities, and the contribution rate of transvariation density to the overall disparities exhibits the most significant increase. (2) The CCD-URBE demonstrates strong stability, as most regions tend to maintain their original CCD-URBE grades. Meanwhile, neighborhood grades moderate the local transition probability significantly. Neighborhoods with high CCD-URBE promote the upward improvement of the local CCD-URBE, while those with low CCD-URBE inhibit it. (3) The CCD-URBE across the nation and the four major regions shows obvious trends of σ-convergence, absolute β-convergence, and conditional β-convergence. The central region, which has lower CCD-URBE, exhibits higher convergence speed. Based on these findings, targeted policy implications are derived.

## 1. Introduction

China is a country with a typical urban–rural dual structure, where urban and rural areas exist almost as two separate worlds. Urban residents generally enjoy higher incomes and have access to superior public services, including education, healthcare, social security, and infrastructure. In stark contrast, rural residents face significantly worse living conditions across nearly all aspects of life [1,2,3]. Over the past 40 years of reform and opening up, China has developed rapidly. Tremendous changes took place in various fields. However, the urban–rural dual structure persists, and urban–rural development remains highly uncoordinated [4,5]. Consequently, “escaping the countryside to seek a better life in urban areas” has been a longstanding dream of almost every generation of Chinese rural residents [6,7]. Since the reform and opening up, education has been the most important way for rural residents to “escape the countryside” [8,9,10]. They improve their academic qualifications and skills via education. Then they engage in non-agricultural occupations or start businesses in cities. Finally, they settle down there. As a result, many rural families in China attach great importance to basic education [11]. There is a common saying in rural areas: “Even if we have to sell everything we own, we will support our children to go to school.” This means rural families are willing to pay any price to ensure their children receive a good basic education. They hope their children can be admitted to universities, especially top universities, through the college entrance examination.

Basic education serves as the cornerstone of the national education system and the starting point for every citizen’s personal growth and success. Its fairness and coordination directly impact individuals’ developmental opportunities, intergenerational mobility, and the overall justice of society [12]. However, due to the urban–rural dual structure and the historical path dependence of policy orientation, the urban and rural basic education systems in China have limited interaction. Driven by industrialization and urbanization, urban basic education has developed at a high speed. In contrast, rural basic education has advanced at an extremely slow pace. The coupled and coordinated development level of urban–rural basic education is not high. (*For brevity, the coupled and coordinated development level of urban–rural basic education is hereafter abbreviated as **CCD-URBE**.*) For a long time, rural residents have been treated unfairly in basic education compared with urban residents [13]. Urban basic education is superior to rural basic education in many aspects. These aspects include teaching staff quality, fund investment, school-running resources, family and social support, and education quality. Correspondingly, urban students have greater opportunities for further education. Their admission rates to high-quality schools are far higher than those of rural students [14]. This unfair phenomenon has become a hot issue in China and has been discussed repeatedly. Against this backdrop, the Chinese government has adopted a series of policies [15]. These policies aim to strengthen the exchange and interaction between urban and rural basic education systems. The government has also increased investment in rural basic education to narrow the development gap with cities. Through these measures, the government intends to promote CCD-URBE, mitigate social conflicts, and achieve greater social equity.

Several fundamental questions therefore arise: What is China’s current CCD-URBE? What kind of dynamic evolution has it experienced over the past few decades? And what patterns can be observed in this process? With ongoing economic and social development, does the CCD-URBE of China exhibit convergence? In other words, will it stabilize at a high development level, and will regions with low CCD-URBE develop to a higher level? If convergence exists, what are the driving factors? If convergence does not exist, what are the major obstacles? Addressing these questions will contribute to a deeper understanding of the status and patterns of CCD-URBE in China. This, in turn, will provide theoretical, empirical, and policy support for optimizing and enhancing this development.

The rest of this paper is organized as follows: Section 2 briefly reviews the relevant literature. Section 3 introduces the empirical methods and data sources. Section 4 reports the measurement results of China’s CCD-URBE and analyzes its evolutionary trends from both temporal and spatial perspectives. Section 5 discusses the convergence of China’s CCD-URBE. Finally, the whole paper is summarized, and corresponding policy implications are put forward.

## 2. Literature Review

Scholars have devoted considerable attention and research to the urban–rural development of basic education. The literature relevant to this study primarily covers two aspects: (1) The development status and trends of urban–rural basic education; and (2) the application and discussion of measurement methods such as the entropy weight method, coupling coordination degree model, and convergence tests.

### 2.1. The Development Status and Trends of Urban–Rural Basic Education

Regarding the development status and trends of urban–rural basic education, existing studies have explored this topic from multiple dimensions, yielding research outcomes that reflect both consensus and divergence.

In terms of urban–rural education development, Sumida and Kawata [16] argued that the main purpose of education is to enhance knowledge and skills. Accordingly, they employed learning performance to assess the level of basic education in sub-Saharan Africa and measured the urban–rural disparity in basic education through the achievement gap. In China, college enrollment serves as a hallmark of basic education outcomes. Zhao [17] posited that college enrollment could, to some extent, reflect the level of basic education in China. His study found that although both urban and rural basic education levels had increased substantially, significant inequalities persisted. Turning to Western Europe, van Maarseveen [18] and Zahl-Thanem and Rye [19] employed higher education attainment rates to evaluate the development of basic education in the Netherlands and Norway, respectively. Both studies concluded that urban–rural coordination in basic education was low, with a widening urban–rural gap.

Viewing basic education as a systemic project, Chen and Zhi [20] contended that its various aspects should be discussed separately. They compared urban and rural investments in teacher resources, information infrastructure, and digital resources in China, and systematically measured the urban–rural levels and disparities in basic education digitalization. Unidimensional characterization and comparison often fail to capture the overall urban–rural status of education. Guo and Li [21] adopted the CRITIC-AHP weighting method to construct a comprehensive evaluation index (EDL). This index covers educational opportunities (e.g., student/teacher ratio, resource allocation) and educational outcomes (e.g., proportion of educated population). They used this index to quantify the development level of urban–rural education. By employing the Education Inequality Index (EII), coefficient of variation (CV), and decomposable Theil index, they quantitatively analyzed China’s urban–rural educational coordination status from 2003 to 2019. Other scholars incorporated factors such as educational resource inputs and educational achievements and employed methods such as PCA and the entropy approach to construct composite indicators for evaluating educational development. These studies yielded valuable research findings [22,23,24].

### 2.2. Application and Discussion of Related Methods

The entropy weight method, coupling coordination degree model, and convergence test are important tools for this study. Existing research has produced abundant results regarding the theory and application of these methods.

#### 2.2.1. Entropy Weight Method

In terms of evaluation and measurement, the entropy weight method is widely used. This is due to its strong objectivity and high operability. Roszkowska and Wachowicz [25] studied the normalization effects of entropy-based weights and Hellwig method. Through a case study on EU educational sustainability, they demonstrated that max-min normalization alters EWM weights but minimally affects final rankings in highly correlated data structures. Zhu et al. [26], however, questioned the limitations of the entropy weight method. They pointed out that the method focused only on the variability of indicators while overlooking their substantive importance, which could lead to distorted measurement results. This argument provided an important reference for subsequent improvements of the entropy method and the integration of subjective and objective weighting methods. Wang et al. [27] introduced the analytic hierarchy process (AHP)-entropy weight method into the evaluation of tourism mascot design. Using six scenic spots in Xi’an, China as a case study, they verified the effectiveness of combined weighting in balancing expert experience and consumer data. In the engineering field, Yang et al. [28] applied the entropy weight method with the objective of optimizing the importance of ventilation network nodes. By using the method to assign weights to evaluation indicators, they identified the importance ranking of all nodes in the network. Fu [29] constructed a curriculum quality evaluation system from the perspective of student perception. By combining objective weighting via the EWM with fuzzy evaluation processing using a cloud model, the empirical study showed that this model can effectively quantify teaching effects and identify dimensions for improvement.

#### 2.2.2. Coupling Coordination Degree Model

The coupling coordination degree model (CCD) serves as a core method for evaluating the synergistic development of multiple systems. Its theoretical foundation is rooted in the quantitative analysis of interactions and dynamic correlations between systems. Specifically, the coupling degree reflects the extent of interaction and correlation between systems, while the coordination degree represents the matching between their developments. The coupling coordination degree, as a comprehensive measure, assesses the overall level of both the interconnectedness and synergistic development of systems [30,31].

Geng et al. [32] developed a comprehensive evaluation system and a CCD model to analyze the coordinated development between higher education and the digital economy in the Yangtze River Economic Belt of China. Their study revealed spatiotemporal evolutionary patterns and identified key influencing factors. Zhang et al. [33] focused on socioeconomic and ecological systems in urban areas. By employing the entropy weight method and the CCD model, they measured the level of coordinated development in 41 cities of the Yangtze River Delta during 2010–2020. Li et al. [34] investigated the Yellow River Basin by constructing a county-level CCD model of production, living, and ecological spaces (PLES). Combined with the geographically weighted regression method, they systematically quantified the spatiotemporal evolution of PLES from 1995 to 2015 and its geographical influencing factors. Some other scholars used the CCD model to study the coordinated relationship between urbanization and ecosystems [35,36]. These valuable explorations have expanded the application scope of the CCD model.

#### 2.2.3. Convergence Test

Research on the convergence issue can be traced back to the neoclassical growth theory. The neoclassical growth model explains the gap in economic growth levels among countries by technological progress. It argues that economic development will eventually tend toward a steady state, which is attributed to the diminishing marginal product of capital. This potential phenomenon is termed the “convergence of economic growth” [37]. Here, convergence includes σ convergence and β convergence. σ convergence refers to the “narrowing of gaps in outcomes,” which measures the reduction in the degree of dispersion. β convergence, on the other hand, captures the “catch-up effect in the process,” indicating that less developed regions exhibit higher growth rates compared to developed regions. Together, these concepts characterize the convergence or divergence features of economic growth [14]. Camalho et al. [38] utilized both σ and β convergence methods to analyze the convergence of education and training systems across European countries. Xiang and Stillwell [23], drawing on Sen’s capability approach, pointed out that the gaps between urban and rural areas in per-student funding and teacher allocation had narrowed in China. However, they argued that the weak educational foundation and limited conversion capability in rural regions prevented the disparities in educational outcomes from converging significantly. Li and Luo [39] applied a β-convergence model to compulsory education data from 31 Chinese provinces. They found evidence of absolute β-convergence at the national level. Yet, the convergence between urban and rural areas proceeded more slowly and did not exhibit significant conditional convergence. Zhang et al. [24] analyzed time-series data from 1995 to 2022 at the national level in China, reaching similar conclusions. Other scholars also employed convergence tests in the fields of economics, environment, and energy, providing valuable insights [40,41,42].

Previous studies have provided a solid foundation and references for this research. However, there are still areas that need further deepening and improvement. First, the measurement of urban and rural basic education development in previous studies was not sufficiently comprehensive or systematic. Most studies focused only on kindergarten or primary school stages. They included school-based resources and outcomes but overlooked the role of families. To address these limitations, this study incorporates all stages of basic education into the evaluation framework. It also includes family engagement and financial investment. This ensures a more comprehensive and systematic evaluation. Second, at present, no research has been found to explore the coupled and coordinated development of urban and rural basic education. However, the urban and rural basic education systems have certain connections and interactions. Their balanced development is essential for social harmony. Therefore, research on measuring the coupled and coordinated development level of China’s urban–rural basic education and its dynamic evolution is of great value. It is also an innovation of this study. Third, previous convergence tests of urban–rural education often overlooked spatial correlation. However, neighboring regions tend to engage in more economic, cultural, and educational exchanges. Spatial dependence is therefore unavoidable. Ignoring it may lead to biased results. To address this issue, this study explicitly considers spatial factors. It employs spatial econometric models to test the convergence of urban–rural coupled and coordinated development in basic education. This makes the test results more reliable.

**Figure 1:**
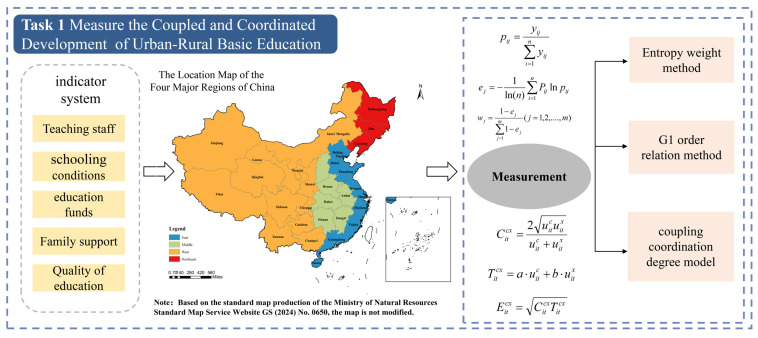

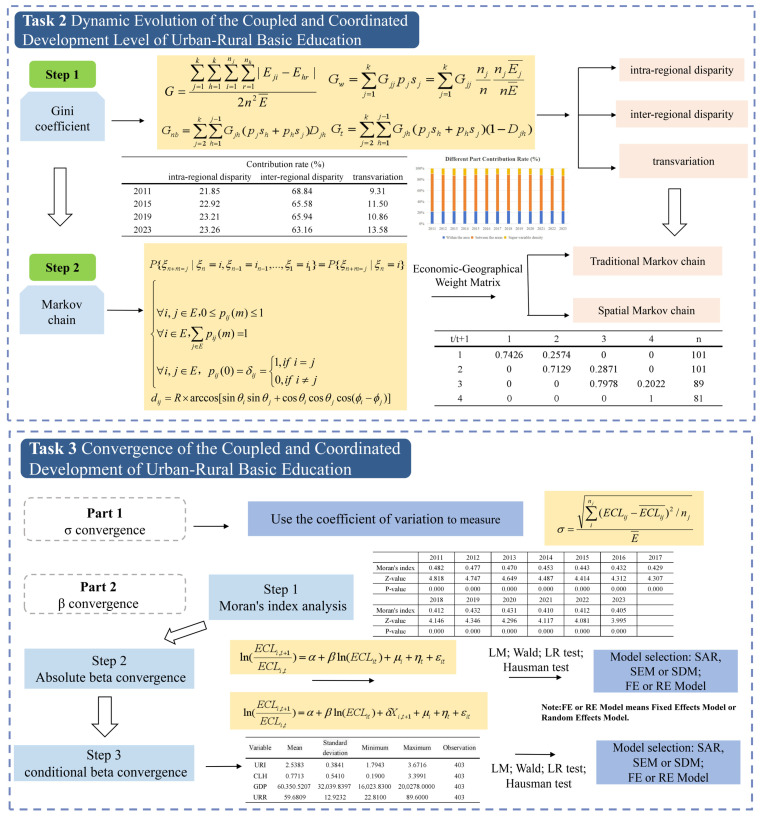
Research Flow Chart.

The main process of this paper is shown in Figure 1.

## 3. Methodology and Data

### 3.1. Evaluation Method for the CCD-URBE

#### 3.1.1. Indicator System

With the advancement of China’s “Education Power” strategy, the development gap between urban and rural basic education has gradually narrowed. The CCD-URBE has also improved. However, structural contradictions still exist in several aspects. These aspects include resource allocation and development balance. With reference to existing studies [21,24], this study focuses on the entire education chain. The chain covers “resource input-process implementation-result output”. The study selects 28 secondary indicators from five dimensions. These five dimensions are teaching staff, school-running conditions, fund investment, family support, and education quality. Details are shown in Table 1. To neutralize the effect of regional population size on absolute indicators and to strengthen cross-regional comparability, all variables are expressed in per capita form.

#### 3.1.2. Evaluation Method

The entropy weight method is an objective weighting method. It assigns weights based on the characteristics of the data itself and has high credibility. However, it may sometimes produce results that contradict the actual importance of indicators [26,27]. The G1 order relation method is a subjective weighting method. First, it ranks indicators according to their importance. Then, it determines the importance ratio between adjacent indicators. Finally, it calculates the weight of each indicator through these ratios. This method relies on the experience of domain experts and can be applied to complex evaluation scenarios [43]. This study adopts a G1–entropy combined weighting method to comprehensively evaluate the CCD-URBE in China from 2011 to 2023. The specific steps are as follows:

Step 1: Conduct standardization processing on each indicator.

(1) $$\begin{array}{c} y_{ij}=\frac{x_{ij}-min{\{x}_{1j},\;x_{2j},\ldots ,x_{nj}\}\;}{max{\{x}_{1j},\;x_{2j},\ldots ,x_{nj}\}-min{\{x}_{1j},\;x_{2j},\ldots ,x_{nj}\}} \end{array}$$

Step 2: Determine indicator weights using the entropy weight method.

(2) $$\begin{array}{c} P_{ij}=\frac{y_{ij}}{\sum_{i=1}^{n}y_{ij}} \end{array}$$

(3) $$\begin{array}{c} e_{j}=-\frac{1}{ln(n)}\sum_{i=1}^{n}p_{ij}lnp_{ij} \end{array}$$

(4) $$\begin{array}{c} w_{j}=\frac{1-e_{j}}{\sum_{j=1}^{m}1-e_{j}} \end{array}$$

Here, *i* represents the number of provinces, with the maximum value of *n*; *j* represents the number of indicators, with the maximum value of *m*.

Step 3: Determine indicator weights using the G1 order relation method.

(5) $$\begin{array}{c} y_{1}>y_{2}>\ldots >y_{m} \end{array}$$

(6) $$\begin{array}{c} r_{i}=\frac{w_{i}}{w_{i+1}} \end{array}$$

(7) $$\begin{array}{c} w_{i}=\frac{\prod_{k=i}^{m-1}r_{i}}{C}\left(i=1,2,\ldots ,m-1\right) \end{array}$$

Here, $y_{i}$ represents various indicators, $w_{i}$ is the weight of the indicator, and C is a normalization constant.

Step 4: Adopt a linear weighted combination model. Let ${w_{o}=(w_{1}^{o},w_{2}^{o},...,w_{m}^{o})}^{T}$ be the weight vector obtained by the entropy weight method. Let ${w_{s}=(w_{1}^{s},w_{2}^{s},...,w_{m}^{s})}^{T}$ be the weight vector obtained by the G1 order relation method.

(8) $$\begin{array}{c} w_{i}^{c}=\alpha \cdot w_{i}^{s}+(1-\alpha )\cdot w_{i}^{o} \end{array}$$

Here, $w_{i}^{c}$ is the final indicator weight.

Step 5: Coupling coordination degree model of urban–rural basic education. The coupling coordination degree model is used to measure the CCD-URBE of each province in China. A larger value of this index indicates better interaction, coordination, and higher balance between urban and rural basic education. On the contrary, a smaller value means disjointed development and lower balance of urban–rural basic education [31,32].

(9) $$\begin{array}{c} {\;Coupling\;Degree:\;C}_{it}^{cx}=\frac{2\sqrt{u_{it}^{c}u_{it}^{x}}}{u_{it}^{c}{+u}_{it}^{x}} \end{array}$$

(10) $$\begin{array}{c} {Coordination\;Degree:\;T}_{it}^{c}=a\cdot u_{it}^{c}+b\cdot u_{it}^{x} \end{array}$$

(11) $$\begin{array}{c} {Coupling\;Coordination\;Degree:\;E}_{it}^{cx}=\sqrt{C_{it}^{cx}T_{it}^{cx}} \end{array}$$

Here, *i* denotes the province and *t* denotes the year. $C_{it}^{cx}$ is the urban–rural coupling degree. $u_{it}^{c}$ and $u_{it}^{x}$ represent the comprehensive indices of basic education development level in urban and rural areas, respectively. *a* and *b* represent the contribution degrees of urban and rural basic education development levels to the coordinated urban–rural development. In this study, *a* = *b* = 0.5 is set. This means the development of urban and rural areas is considered equally important for the coupling coordination development of urban–rural basic education.

There is no consensus in the academic community on the classification of coupling coordination degree. With reference to previous studies and combined with the actual conditions of various regions in China [32,33,34,35], we have divided the coupling coordination degree into 6 types, and the classification results are shown in Table 2.

To maintain consistency in the weight of each indicator in the urban and rural basic education evaluation systems, the following steps are taken first: calculate the weight value of each indicator based on all indicators at the provincial level. Then, compute the comprehensive evaluation index of basic education development level separately using the specific data of each urban and rural indicator.

In terms of regional classification, China’s economic regions are divided into four major regions: Eastern, Central, Western, and Northeast China. This division is based on the Method for Dividing Eastern, Central, Western and Northeast China issued by the National Bureau of Statistics of China (Accessed from: https://www.stats.gov.cn/zt_18555/zthd/sjtjr/dejtjkfr/tjkp/202302/t20230216_1909741.htm (accessed on 1 September 2025)). Specifically, the Eastern region includes 10 provinces and municipalities directly under the Central Government: Beijing, Tianjin, Hebei, Shanghai, Jiangsu, Zhejiang, Fujian, Shandong, Guangdong, and Hainan. The Central region includes 6 provinces: Shanxi, Anhui, Jiangxi, Henan, Hubei, and Hunan. The Western region includes 12 provinces, autonomous regions, and municipalities directly under the Central Government: Inner Mongolia, Guangxi, Chongqing, Sichuan, Guizhou, Yunnan, Tibet, Shaanxi, Gansu, Ningxia, Qinghai, and Xinjiang. The Northeast region includes 3 provinces: Liaoning, Jilin, and Heilongjiang.

### 3.2. Regional Disparity Decomposition

To understand the regional disparity in the CCD-URBE, this study adopts the Gini coefficient decomposition method. The Dagum Gini coefficient decomposition method is a significant extension of the Gini coefficient proposed by Corrado Gini [44,45], and was systematically refined by Christian Dagum in 1997 [46,47,48,49]. This method is used to decompose the regional disparity of the coupling coordination development level [14,50]. The formula for the Gini coefficient is as follows:

(12) $$\begin{array}{c} G=\frac{\sum_{j=1}^{k}\sum_{h=1}^{k}\sum_{i=1}^{n_{j}}\sum_{r=1}^{n_{h}}\left|E_{ji}-E_{hr}\right|}{2n^{2}\bar{E}} \end{array}$$

Here, G represents the Gini coefficient. j and h represent the number of regions. i and r represent the number of provinces within a region. k represents the total number of regions. n represents the total number of provinces. $n_{j}(n_{h})$ represents the number of provinces in the *j*(*h*)-th region. $E_{ji}(E_{hr})$ represents the CCD-URBE of province *i*(*r*) in the *j*(*h*)-th region. $\bar{E}$ represents the arithmetic mean of the CCD-URBE.

When conducting decomposition, the following steps are required first: sort the *k* regions according to the mean value of the CCD-URBE within each region. Then, decompose the Gini coefficient G into three parts: the contribution of intra-regional disparities ($G_{w}$), the contribution of inter-regional disparities ($G_{nb}$), and the contribution of transvariation density ($G_{t}$). And the equation $G=G_{w}+G_{nb}+G_{t}$ holds. The specific formulas are as follows:

(13) $$\begin{array}{c} G_{jj}=\frac{\frac{1}{2{\bar{E}}_{j}}\sum_{i=1}^{n_{j}}\sum_{r=1}^{n_{h}}|E_{ji}-E_{hr}|}{n_{j}^{2}} \end{array}$$

(14) $$\begin{array}{c} G_{w}=\sum_{j=1}^{k}G_{jj}p_{j}s_{j}=\sum_{j=1}^{k}G_{jj}\frac{n_{j}}{n}\frac{n_{j}{\bar{E}}_{j}}{n\bar{E}} \end{array}$$

(15) $$\begin{array}{c} G_{jh}=\sum_{i=1}^{n_{j}}\sum_{r=1}^{n_{h}}\frac{\left|E_{ji}-E_{hr}\right|}{n_{j}n_{h}({\bar{E}}_{j}+{\bar{E}}_{h})} \end{array}$$

(16) $$\begin{array}{c} G_{nb}=\sum_{j=2}^{k}\sum_{h=1}^{j-1}G_{jh}(p_{j}s_{h}+p_{h}s_{j})D_{jh} \end{array}$$

(17) $$\begin{array}{c} G_{t}=\sum_{j=2}^{k}\sum_{h=1}^{j-1}G_{jh}(p_{j}s_{h}+p_{h}s_{j})(1-D_{jh}) \end{array}$$

(18) $$\begin{array}{c} D_{jh}=\frac{d_{jh}-p_{jh}}{d_{jh}+p_{jh}} \end{array}$$

(19) $$\begin{array}{c} d_{jh}=\int_{0}^{\infty }dF_{j}(E)\int_{0}^{E}F_{j}(E-x)dF_{h}(x) \end{array}$$

(20) $$\begin{array}{c} p_{jh}=\int_{0}^{\infty }dF_{h}(E)\int_{0}^{E}F_{j}(E-x)dF_{j}(x) \end{array}$$

Here, $G_{jj}$ denotes the Gini coefficient within Region j, while $G_{jh}$ denotes the Gini coefficient between Region j and Region h. $D_{jh}$ indicates the relative impact of the CCD-URBE between Region j and Region h. $d_{jh}$ represents the disparity in the CCD-URBE between regions, defined as the expected value of the sum of all sample values where $E_{ji}-E_{hr}>0$ in Region j and h. $P_{jh}$ represents the first-order moment of transvariation, which is likewise defined as the expected value of the sum of all sample values where $E_{hr}-E_{ji}>0$ in Region j and h. Finally, $F_{j}(F_{h})$ denotes the cumulative distribution function of the CCD-URBE in Region *j(h)*.

### 3.3. Dynamic Evolution Trend

The Markov chain method provides a good tool for studying the dynamic evolution of the CCD-URBE.

Both the traditional Markov chain and the spatial Markov chain are non-parametric estimation methods. Without requiring assumptions about distributional forms, they evaluate the performance of estimators based on the information contained in random samples. As a result, the estimation outcomes are more robust and reliable [51,52]. The traditional Markov chain discretizes data into *S* types. Under the condition of discrete time and state, it calculates the probability distribution and evolution trend of each type. Thus, it reveals the development characteristics of CCD-URBE at different levels. Let $\left\{\left.\xi_{n},n=1,2,\ldots \right\}\right.$ be a random sequence. The state space *H* represents the set of all possible values of each random variable in the random sequence. For any positive integers *m* and *n*, and for $i,j,i_{k}\in H\;(where\;H=\{k=1,\ldots ,n-1\})$, we have:

(21) $$\begin{array}{c} P\{\xi_{n+m}=j\;|\xi_{n}=i,\xi_{n-1}=i_{n-1},\ldots ,\xi_{1}=i_{1}\}=P\{\xi_{n+m}=j|\xi_{n}=i\} \end{array}$$

Then $\left\{\left.\xi_{n},n=1,2,\ldots \right\}\right.$ is called a Markov chain, and the above formula is referred to as the Markov property. For a Markov chain $\left\{\left.\xi_{n},n=1,2,\ldots \right\}\right.$, the matrix $p(m)=(P_{ij}(m))$ with the *m*-step transition probability $P_{ij}(m)$ as its elements is the *m*-step transition matrix of this chain. When *m* = 1, it is simply called the transition matrix, which has the following properties:

(22) $$\begin{array}{c} \left\{\begin{array}{l} \forall i,j\in H,0\leq p_{ij}(m)\leq 1\\ \forall i\in H,\sum_{j\in E}p_{ij}(m)=1\\ \forall i,j\in H,\;p_{ij}(0)=\delta_{ij}=\left\{\begin{array}{ll} 1, & if\;i=j\\ 0, & if\;i\neq j \end{array}\right. \end{array}\right. \end{array}$$

The spatial Markov chain model introduces the concept of “spatial lag” and examines how spatial factors, that is, the neighboring regions, influence the transition probabilities of a given region. To measure the degree of influence between regions, a spatial weight matrix is required. Since the development of basic education is not only related to geographical distance but also affected by economic development, this study comprehensively considers both factors and adopts an economic–geographical weight matrix $W_{ij}$ [44,45]. $W_{ij}$ is a 31 × 31 matrix, where all diagonal elements are 0. The off-diagonal elements represent the standardized weights of economic–geographical distance, which reflect the strength of the association between province *i* and province *j*. The stronger the association, the larger the weight value.

(23) $$\begin{array}{c} d_{ij}=R\times \arccos[\sin\theta_{i}\sin\theta_{j}+\cos\theta_{i}\cos\theta_{j}\cos(\phi_{i}-\phi_{j})] \end{array}$$

(24) $$\begin{array}{c} w_{ij}^{g}=\left\{\begin{array}{ll} \frac{1}{d_{ij}}, & i\neq j\\ 0, & i=j \end{array}\right. \end{array}$$

(25) $$\begin{array}{c} w_{ij}^{e}=\left\{\begin{array}{ll} \frac{1}{\bar{y_{i}}-\bar{y_{j}}}, & i\neq j\\ 0, & i=j \end{array}\right. \end{array}$$

(26) $$\begin{array}{c} W_{ij}=w_{ij}^{g}\cdot w_{ij}^{e} \end{array}$$

Here, R represents the radius of the Earth. $\theta_{i}$ and $\theta_{j}$ represent the latitudes (in radians) of province *i* and province *j*, respectively. $\phi_{i}$ and $\phi_{j}$ represent the longitudes (in radians) of province *i* and province *j*, respectively. The conversion formula from degrees to radians is: Radians = Degrees × (π/180). $\bar{y_{i}}$ and $\bar{y_{j}}$ represent the total per capita GDP of the *i*-th and *j*-th province during the study period, respectively.

For an *S* × *S* Markov transition matrix, when *T* types of spatial lag are considered as transition conditions, *T* transition matrices (each of size *S* × *S*) will be generated, denoted as $P_{ij|\varphi }^{t,t+d}$. $P_{ij|\varphi }^{t,t+d}$ represents the probability that the CCD-URBE in the local region transfers from type i to type j after d years, under the condition that the spatial lag of CCD-URBE in the current year is of type φ. Changes in the CCD-URBE that occur between adjacent types are referred to as upward or downward transitions. Changes that cross non-adjacent types are called positive or negative leapfrog transitions. By comparing the values of the transition matrices of the traditional Markov chain and the spatial Markov chain, it can be determined whether neighboring provinces have an impact on the transition process of the CCD-URBE. If for all i and j, the condition $P_{ij|\varphi }^{t,t+d}$ = $P_{ij}^{t,t+d}$ holds, there is no spatial spillover effect among provinces in terms of CCD-URBE. Neighboring provinces at any level will not affect the transition of the CCD-URBE in the local province.

### 3.4. Convergence

With the continuous improvement of China’s urban–rural relations, the CCD-URBE is likely to converge toward a certain steady state. Therefore, it is highly appropriate to use a convergence test model to study the long-term variation characteristics of the CCD-URBE.

#### 3.4.1. σ-Convergence

σ-convergence reflects whether the deviations of the CCD-URBE across different regions decrease over time. In this study, the coefficient of variation (CV) is used to measure the σ-convergence of the CCD-URBE [14,38]. The calculation formula is as follows:

(27) $$\begin{array}{c} \sigma =\frac{\sqrt{\sum_{i}^{n_{j}}(ECL_{ij}-\bar{ECL_{ij}}{)}^{2}/n_{j}}}{\bar{E}} \end{array}$$

Here, ${ECL}_{ij}$ (Educational Coordination Level) represents the CCD-URBE of province *i* within region *j*, $\bar{{ECL}_{ij}}$ denotes the average value of the CCD-URBE within region *j*, and $n_{j}$ stands for the number of provinces within region *j*.

#### 3.4.2. β-Convergence

β-convergence refers to the phenomenon where, as time progresses, less developed regions exhibit a higher growth rate to catch up with more developed regions. The gap between the two gradually narrows, and they eventually reach the same steady-state level. β-convergence can be further categorized into absolute β-convergence and conditional β-convergence. Absolute β-convergence means that the CCD-URBE tends to converge across regions, without considering a series of factors that have a significant impact on the CCD-URBE [14,24,38]. The absolute β-convergence model is as follows:

(28) $$\begin{array}{c} \ln\left(\frac{ECL_{i,t+1}}{ECL_{i,t}}\right)=\alpha +\beta \ln(ECL_{it})+\mu_{i}+\eta_{t}+\varepsilon_{it} \end{array}$$

Here, ${ECL}_{i,t+1}$ represents the CCD-URBE of the *i*-th province in period *t* + 1; ${ECL}_{i,t}$ represents the CCD-URBE of the *i*-th province in period *t*; $ln\frac{{ECL}_{i,t+1}}{{ECL}_{i,t}}$ represents the growth rate of the CCD-URBE of the *i*-th province in period *t* + 1; β is the convergence coefficient: if β < 0, it indicates that the CCD-URBE across regions has a convergent trend; on the contrary, if β > 0, there is a divergent trend; $\mu_{i},\;\eta_{t}\;\mathrm{and}\;\varepsilon_{it}$ represent the individual effect, time effect, and random disturbance term, respectively. Define the convergence speed as follows:

(29) $$\begin{array}{c} v=\frac{-\ln(1-|\beta |)}{T} \end{array}$$

There may be spatial correlation in the CCD-URBE. Common spatial econometric models include the Spatial Durbin Model (SDM), Spatial Autoregressive Model (SAR), Spatial Error Model (SEM), and so on. The SDM can be regarded as a general form of the SAR model and the SEM. This study uses panel data, so it is necessary to introduce a spatial panel data model to test the convergence. The spatial absolute β convergence model is as follows:

(30) $$\begin{array}{c} SAR:\ln\left(\frac{ECL_{i,t+1}}{ECL_{i,t}}\right)=\alpha +\beta \ln(ECL_{i,t})+\rho \sum_{j=1}^{n}w_{ij}\ln\left(\frac{ECL_{j,t+1}}{ECL_{j,t}}\right)+\mu_{i}+\eta_{t}+\varepsilon_{it} \end{array}$$

$$\mathrm{SEM}:\ln\left(\frac{ECL_{i,t+1}}{ECL_{i,t}}\right)=\alpha +\beta \ln(ECL_{i,t})+\mu_{i}+\eta_{t}+\mu_{it}$$

(31) $$\begin{array}{c} \mu_{it}=\lambda \sum_{j=1}^{n}w_{it}u_{jt}+\varepsilon_{it} \end{array}$$

(32) $$\begin{array}{c} \mathrm{SDM}:\ln\left(\frac{ECL_{i,t+1}}{ECL_{i,t}}\right)=\alpha +\beta \ln(ECL_{i,t})+\rho \sum_{j=1}^{n}w_{ij}\ln\left(\frac{ECL_{j,t+1}}{ECL_{j,t}}\right)+\gamma \sum_{j=1}^{n}w_{ij}\ln(ECL_{j,t})+\mu_{i}+\eta_{t}+\varepsilon_{it} \end{array}$$

Here, ρ is the spatial lag coefficient. It represents the impact of the growth rate of the CCD-URBE in neighboring provinces on the current province. λ is the spatial error coefficient. It represents the spatial effect existing in the random disturbance term. γ is the spatial lag coefficient of the independent variable. It represents the impact of the CCD-URBE in neighboring provinces. For the selection of models, the general steps are as follows: First, establish a general panel regression model. Then use the LM statistic to test for spatial autocorrelation. If spatial autocorrelation exists, at least one of the SAR and SEMs is valid. Second, establish the SDM. Then use the Wald statistic and LR statistic to determine whether it can be simplified to SAR or SEM [53]. To avoid endogenous interference of economic and social distance on the model, this study sets the weight in the spatial weight matrix as the reciprocal of the square of geographical distance. This is different from the economic–geographical weight matrix constructed earlier.

(33) $$\begin{array}{c} w_{ij}=\left\{\begin{array}{ll} \frac{1}{d_{ij}^{2}}, & i\neq j\\ 0, & i=j \end{array}\right. \end{array}$$

The conditional β convergence model extends the absolute β convergence model by including a series of control variables. It examines whether, after controlling for a set of factors that significantly affect the CCD-URBE, the regions’ CCD-URBE still exhibit a convergence trend. The conditional β convergence model is specified as follows [14,24,38]:

(34) $$\begin{array}{c} \ln\left(\frac{ECL_{i,t+1}}{ECL_{i,t}}\right)=\alpha +\beta \ln(ECL_{i,t})+\delta X_{i,t+1}+\mu_{i}+\eta_{t}+\varepsilon_{it} \end{array}$$

(35) $$\begin{array}{c} \mathrm{SAR}:\ln\left(\frac{ECL_{i,t+1}}{ECL_{i,t}}\right)=\alpha +\beta \ln(ECL_{it})+\rho \sum_{j=1}^{n}w_{ij}\ln\left(\frac{ECL_{j,t+1}}{ECL_{j,t}}\right)+\delta X_{i,t+1}+\mu_{i}+\eta_{t}+\varepsilon_{it} \end{array}$$

$$SEM:\ln\left(\frac{ECL_{i,t+1}}{ECL_{i,t}}\right)=\alpha +\beta \ln(ECL_{i,t})+\delta X_{i,t+1}+\mu_{i}+\eta_{t}+\mu_{it}$$

(36) $$\begin{array}{c} \mu_{it}=\lambda \sum_{j=1}^{n}w_{it}u_{jt}+\varepsilon_{it} \end{array}$$

(37) $$\begin{array}{c} \mathrm{SDM}:\ln\left(\frac{ECL_{i,t+1}}{ECL_{i,t}}\right)=\alpha +\beta \ln(ECL_{i,t})+\rho \sum_{j=1}^{n}w_{ij}\ln\left(\frac{ECL_{j,t+1}}{ECL_{j,t}}\right)+\gamma \sum_{j=1}^{n}w_{ij}\ln(ECL_{j,t})+\delta X_{i,t+1}+\mu_{i}+\eta_{t}+\varepsilon_{it} \end{array}$$

Here, *X*_*i*,*t*+1_ represents a series of control variables that affect the CCD-URBE. δ is its coefficient vector.

With reference to existing studies, the selection of control variables should fully consider the main factors affecting the development of basic education [24,39]. Economic level is the core support for basic education resource input. The level of economic development can be directly transformed into the supply capacity of basic education. Therefore, economic level is included as a control variable, measured by per capita GDP. The urban–rural income gap can indirectly affect the CCD-URBE through the “affordability of quality education resources”. Thus, the urban–rural income gap (*URI*) is selected as a control variable, measured by the ratio of urban to rural per capita income. The core production factors of basic education directly determine teaching quality. Hence, social public resources (*CLH*) are chosen as a control variable, measured by the per capita book collection. Urbanization affects the supply-demand structure of urban–rural basic education through population mobility. So urbanization needs to be controlled, measured by the urbanization rate (*URR*). The descriptive statistics of the control variables are shown in Table 3.

All programming and data analysis were performed using MATLAB 2024a and Stata 18.

### 3.5. Data Sources

The data used in this study are provincial-level data from 31 provinces in mainland China during 2011–2013. Data on various indicators of teacher resources and school-running conditions are sourced from the Educational Statistics Yearbook of China. Data on various indicators of educational funds are obtained from the China Educational Finance Statistical Yearbook. Data such as per capita educational consumption expenditure, per capita GDP, per capita book collection, and urbanization rate are derived from two sources. One source is the statistical yearbooks of various provinces in China. The other source is the China National Economic and Social Development Statistical Bulletin. Data on average years of schooling come from two publications. They are China Statistical Yearbook and China Population and Employment Statistical Yearbook. The per capita family attention to education is measured in a specific way. It uses the annual per capita number of Baidu searches by urban and rural residents in China during the study period. The search keywords include “enrollment policy, teaching materials, curriculum reform, school district housing, homework load, primary school, junior high school, senior high school, college entrance examination, compulsory education”. Individual missing data are supplemented using two methods. These methods are the moving average method and the trend prediction method. All nominal data are adjusted to real data. The base period for adjustment is 2011.

## 4. The Evolution of CCD-URBE: Across Time and Regions

### 4.1. Measurement Results

**Figure 2:**
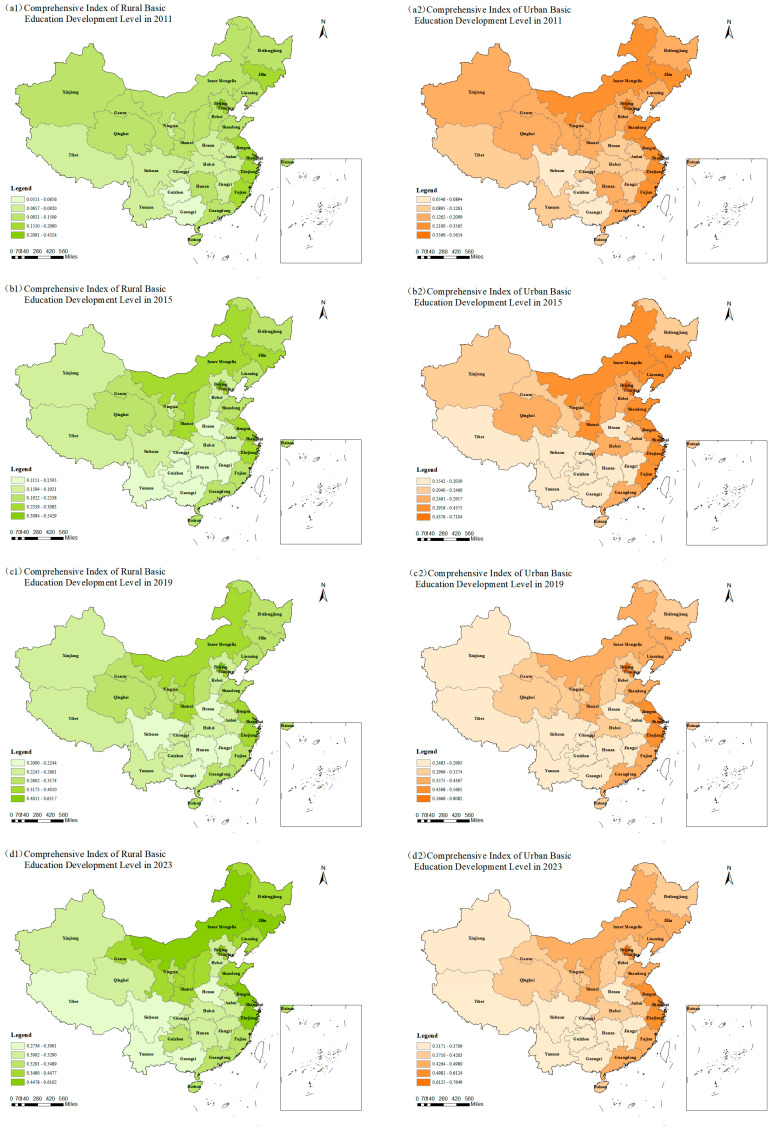
Spatial Pattern of Urban–Rural Basic Education Development in China from 2011 to 2023. Note: This map is made based on the standard map GS (2024) No. 0650 from the Standard Map Service Website of the Ministry of Natural Resources of China. No modifications have been made to the base map.

To reveal the evolution trend of CCD-URBE in China during the study period, this study takes two key steps. First, weights are assigned to each secondary indicator based on the entropy weight method and G1-method. Then, the combined weighting method is used. With this method, the comprehensive index of urban and rural basic education development level for each year during the study period is calculated. The indices of 2011, 2015, 2019, and 2023 are selected to draw the spatial pattern map of urban–rural basic education development level. The map is shown in Figure 2.

Three key findings are obtained from the map. First, from an overall perspective, the comprehensive index of basic education development level of all provinces shows an upward trend during the study period. All provinces make certain progress in basic education. They also achieve significant results in educational resource investment and education quality improvement. Second, the comprehensive index of basic education development level in cities is generally higher than that in rural areas. This reflects that cities have greater advantages over rural areas in educational resource aggregation and teaching staff. The imbalance in urban–rural basic education development still exists. Finally, differences exist in the comprehensive index of basic education development level among different provinces. Among cities, developed regions like Beijing and Shanghai have a higher basic education level. In contrast, relatively underdeveloped regions such as Guizhou have a lower basic education level. The same situation is found in rural areas. This shows that regional economic development level has a great impact on basic education. Economically developed regions can provide more support for basic education. This is basically consistent with the findings of Guo and Li [21] and Zhang et al. [24].

**Figure 3:**
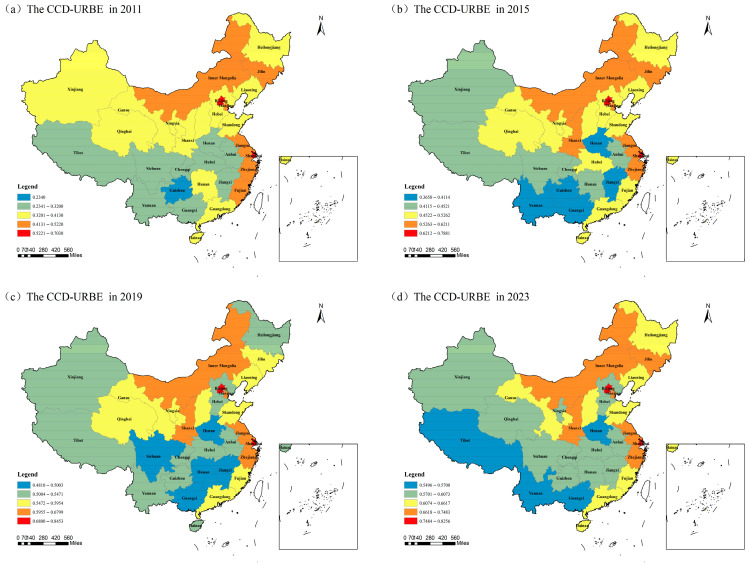
Spatial Pattern of the Coupling Coordination Level of Urban–Rural Basic Education in China from 2011 to 2023. Note: This map is made based on the standard map GS (2024) No. 0650 from the Standard Map Service Website of the Ministry of Natural Resources of China. No modifications have been made to the base map.

Subsequently, a coupling coordination degree model was constructed in this study. This model was used to calculate the CCD-URBE index for each province in each year during the study period. Four typical years (2011, 2015, 2019, and 2023) were selected to present the results. The results are shown in Figure 3. Specifically, two key findings are observed: First, significant differences exist in CCD-URBE among different provinces. Developed regions like Beijing and Shanghai have relatively high CCD-URBE. These regions hold certain advantages in the allocation of urban–rural basic education resources and the improvement of education quality. Thus, the urban–rural gap in these regions is relatively small. In contrast, regions such as Guangxi and Guizhou have relatively low CCD-URBE. Large gaps still exist between urban and rural areas in these regions in terms of educational resources and teaching staff. Second, the growth rates of the CCD-URBE index vary across provinces. Some provinces (e.g., Guizhou, Chongqing, Anhui) have experienced a large increase in the index during the study period. This indicates that these regions have achieved significant results in promoting the CCD-URBE. It also shows that they have accelerated the process of urban–rural education integration. However, some provinces (e.g., Beijing, Tianjin, Fujian) have experienced relatively small growth in the index. These provinces need to explore more refined and innovative development models.

**Figure 4:**
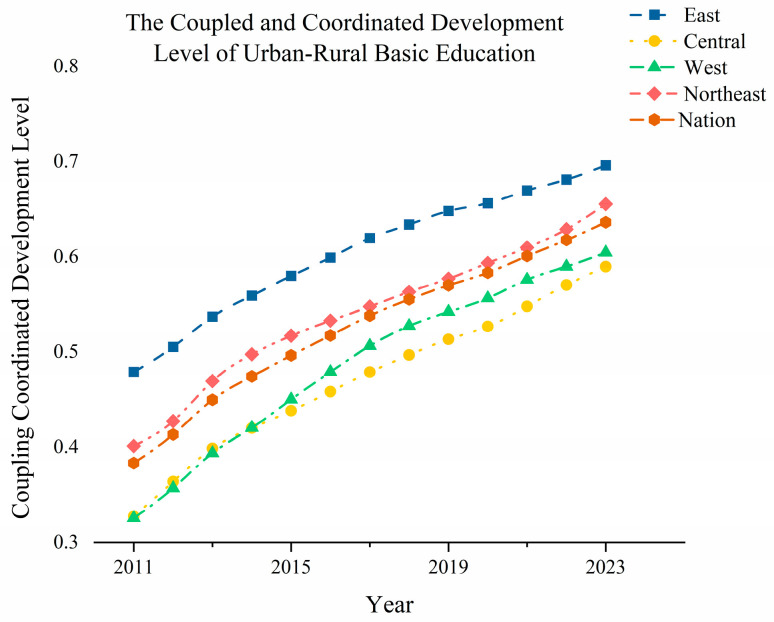
Evolution Trend of CCD-URBE Index in China and Its Regions (2011–2023).

To further study the development of CCD-URBE in different regions, this study analyzed the CCD-URBE at the national level and in the four major regions. It calculated the average value of the CCD-URBE index for the whole country and each region during the study period. Then, it plotted the evolution trend of the CCD-URBE at the national and regional levels, as shown in Figure 4. According to the results, the evolution of China’s CCD-URBE shows the following characteristics: (1) The national CCD-URBE index shows a steady upward trend. It increased steadily from 0.383 in 2011 to 0.637 in 2023. This indicates that, in general, the exchange and interaction between China’s urban and rural basic education systems have increased significantly. The level of balanced development has improved remarkably, and the CCD-URBE has been significantly enhanced during the study period. The continuously promoted balanced development strategy of basic education has achieved positive results. (2) The absolute differences in the CCD-URBE index among the four major regions are relatively obvious, but they tend to narrow over time. The range narrowed from 0.154 in 2011 to 0.106 in 2023. Specifically, the CCD-URBE index of the eastern region has always been the highest, far higher than that of other regions. The CCD-URBE indexes of the central and western regions are relatively close, and that of the northeast region is in the middle. This shows that China’s CCD-URBE has certain characteristics of regional imbalance. The eastern region leads in this aspect due to its high economic development level and abundant educational resources. However, the gaps between regions are gradually narrowing. (3) There are differences in the growth rate of the CCD-URBE index among the four major regions. Specifically, the eastern region achieved steady growth: its index increased from 0.479 in 2011 to 0.696 in 2023, with an average annual growth rate of about 1.67%. The central region’s index rose from 0.327 in 2011 to 0.590 in 2023, with an average annual growth rate of about 2.02%. The western region’s index went up from 0.326 in 2011 to 0.605 in 2023, with an average annual growth rate of about 2.15%. The northeast region’s index increased from 0.401 in 2011 to 0.656 in 2023, with an average annual growth rate of about 1.96%. This shows that the CCD-URBE in all four major regions has been continuously improved. The balance of regional urban–rural basic education is constantly strengthening. The central and western regions have grown relatively faster and are constantly catching up with the eastern region, while the northeast region also maintains a stable growth trend.

To further reflect the spatiotemporal differences of CCD-URBE within regions, this study selected 10 provinces and municipalities directly under the central government in the eastern region as examples for research. Analysis reveals that the CCD-URBE within the eastern region exhibits the following characteristics:

(1) During the study period, the temporal evolution trend of CCD-URBE in all provinces showed a narrowing trend. The average annual growth rates of CCD-URBE in Beijing, Tianjin, Hebei, Shanghai, and Jiangsu were about 1.32%, 2.53%, 3.51%, 2.39%, and 3.61% respectively. The narrowing ranges of the gap were about 17.03%, 34.88%, 51.34%, 32.72%, and 52.97%. The average annual growth rates of CCD-URBE in Zhejiang, Fujian, Shandong, Guangdong, and Hainan were about 3.47%, 3.16%, 4.00%, 4.33%, and 4.80% respectively. The narrowing ranges of the gap were about 50.66%, 45.28%, 60.11%, 66.40%, and 75.47%. During the study period, all provinces in the eastern region made a certain degree of positive progress in narrowing the gap between urban and rural basic education.

(2) During the study period, there were obvious spatial differences in CCD-URBE in the eastern region. The CCD-URBE of each province in descending order was: Beijing, Shanghai, Tianjin, Zhejiang, Jiangsu, Shandong, Fujian, Guangdong, Hebei, Hainan. Among them, the gap of CCD-URBE in Beijing and Shanghai was relatively small. The average values of CCD-URBE during the study period were about 0.80 and 0.74 respectively. As important economic and cultural centers in China, these two municipalities have relatively strong economic development levels and government financial capabilities. They also have certain advantages in the CCD-URBE. On the whole, other provinces are also making continuous efforts to narrow the gap, showing a good development trend.

### 4.2. Overall Disparities and Intra-Regional Disparities

**Figure 5:**
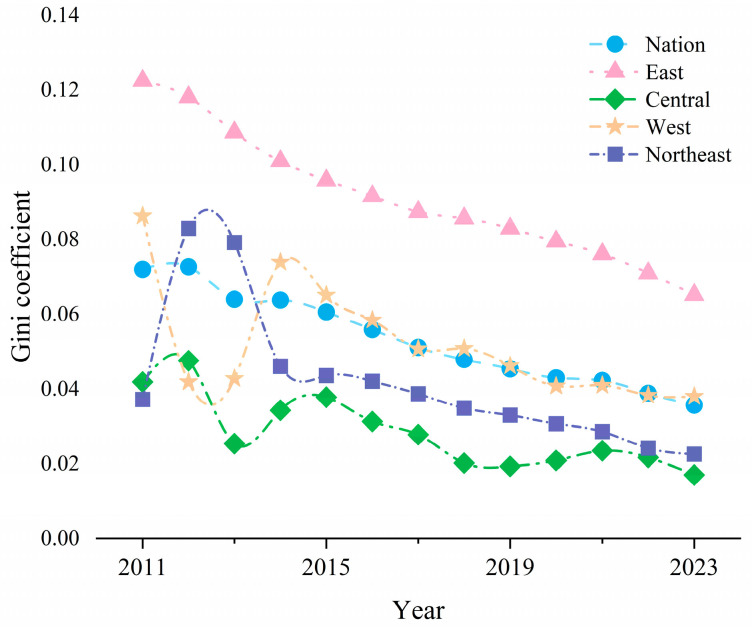
Overall Disparities and Intra-Regional Disparities of CCD-URBE (2011–2023).

Through the above calculation and analysis, the overall spatio-temporal situation of the CCD-URBE has been preliminarily grasped. However, the presentation of a single province only reflects the individual development situation and cannot reveal the degree of disparity and evolution trend in the CCD-URBE among the four major regions of China and among the provinces within each region. Therefore, based on the measurement results, this section further focuses on regional disparities and uses the Dagum Gini coefficient for analysis. The Gini coefficient can accurately quantify the disparities within and between areas. This makes it possible to more scientifically identify the main sources of disparities in CCD-URBE among provinces in China [51]. Figure 5 reports the overall disparities and intra-regional disparities of CCD-URBE in China from 2011 to 2023.

(1) In terms of overall disparities, the overall Gini coefficient of China’s CCD-URBE remained between 0.059 and 0.131 during the study period. This indicates that the overall imbalance of CCD-URBE at the national level is weak. From the perspective of evolution trend, the overall Gini coefficient showed a continuous and steady downward trend. It decreased from 0.131 in 2011 to 0.059 in 2023, with a decrease of 54.96% and an average annual decline rate of about 4.23%. This fully shows that during the study period, the disparity of CCD-URBE across the country was constantly narrowing. The spatial balance of CCD-URBE continued to strengthen.

(2) Specifically, from 2011 to 2015, the Gini coefficient decreased from 0.131 to 0.097. This period was a rapid phase of overall disparity narrowing, with a decrease of 25.95% in 4 years. This may be closely related to China’s policies during the “12th Five-Year Plan” period, such as increasing investment in rural compulsory education and promoting the balanced development of compulsory education. From 2016 to 2023, it entered a “slow narrowing phase”. The Gini coefficient decreased from 0.090 to 0.059, with a decrease of 34.44% in 8 years. Although the decline rate slowed down, it still maintained a stable downward trend. The reasons may be as follows: On the one hand, China continued to tilt resources to rural areas and weak schools through measures such as comprehensively reforming the funding guarantee for urban–rural compulsory education. This narrowed the gap in hardware and teaching staff between urban and rural areas, thereby improving the CCD-URBE. On the other hand, the regional coupled and coordinated development strategy promoted the radiation of high-quality educational resources from the eastern region to the central, western and northeastern regions. This indirectly alleviated the imbalance in basic education development between regions.

Figure 5 also reports the intra-regional disparities of CCD-URBE and their evolution trends.

(1) In terms of the absolute value of disparities, the intra-regional disparities of CCD-URBE among the four major regions showed significant stratification during the study period. The specific performance was “the eastern region is the highest, the western region is the second, and the central and northeastern regions are relatively low”. Specifically, the average intra-regional Gini coefficient of the eastern region was 0.09. This indicates that the disparities in CCD-URBE among provinces within this region are the most prominent. This may stem from the significant disparities in the economic development gradient within the eastern region. Economically developed areas such as Beijing and Shanghai have strong government financial capabilities. They can achieve efficient and balanced allocation of urban–rural basic education resources, resulting in high CCD-URBE. However, provinces such as Hebei and Hainan still have problems of insufficient basic education resources in some rural areas. This leads to obvious disparity in CCD-URBE among provinces within the region. The intra-regional Gini coefficient of the western region was higher than that of the central and northeastern regions in most years. This may be because the western region has a vast territory. The natural conditions and economic development levels of different provinces within the region vary greatly. The imbalance in the allocation of urban–rural basic education resources is further amplified, resulting in relatively high imbalance of CCD-URBE within the region. The average intra-regional Gini coefficient of the northeastern region was 0.04. As an old industrial base, it has a relatively solid foundation for the development of urban–rural basic education within provinces. The intra-regional disparities of CCD-URBE are at a medium level. The intra-regional Gini coefficient of the central region is the lowest among the four major regions. This may be because provinces in this region have a high proportion of agricultural population and great demand for rural basic education. China has provided relatively strong policy support in this region. Therefore, the intra-regional disparity is relatively gentle, and the education development of provinces within the region has a certain synergy.

(2) From the perspective of evolution trends, the intra-regional disparities of CCD-URBE in all four major regions showed a continuous downward trend. But there were obvious differences in the extent and speed of the decline. The balanced development process of urban–rural basic education within different regions had certain heterogeneity. From 2011 to 2015, the intra-regional disparities of CCD-URBE in northeastern region and western region showed an inverted U-shape and a positive U-shape, respectively. This was closely related to the economic and social development trends and policy orientations of the two regions in specific periods.

In the western region from 2011 to 2013, the state continuously increased its educational support to the region. A large amount of special educational funds was invested in the west. The hardware facilities of rural schools were greatly improved, narrowing the gap with urban schools. From 2013 to 2015, as the economy of some western regions gradually developed, the agglomeration effect of cities on educational resources began to become prominent. Cities attracted more high-quality teachers with better economic environment, living conditions and career development opportunities. Some excellent rural teachers also moved to cities. In addition, parents of rural students with better economic conditions chose to send their children to urban schools for better education. This further aggravated the imbalance in the distribution of urban–rural educational resources, leading to the re-expansion of intra-regional disparities in CCD-URBE.

In the northeastern region, it faced great pressure of economic restructuring in the early stage. Some cities concentrated resources to develop advantageous schools in order to improve education quality. They tilted educational funds allocation and teacher deployment towards key urban schools. At the same time, the acceleration of urbanization led to a large number of rural people moving to cities. The surge in student enrollment in urban schools prompted more resources to flow to urban education. The proportion of educational resources in rural schools relatively decreased, and the urban–rural education gap widened rapidly, with the Gini coefficient rising. After 2013, as the state attached more importance to education equity, the northeastern region adjusted its educational resource allocation strategy. The government increased investment in rural basic education and implemented a series of measures to improve the school-running conditions of rural schools. These measures gradually improved the education quality of rural schools, narrowed the disparity in basic education between urban and rural areas again, and the Gini coefficient decreased accordingly.

### 4.3. Inter-Regional Disparities

**Figure 6:**
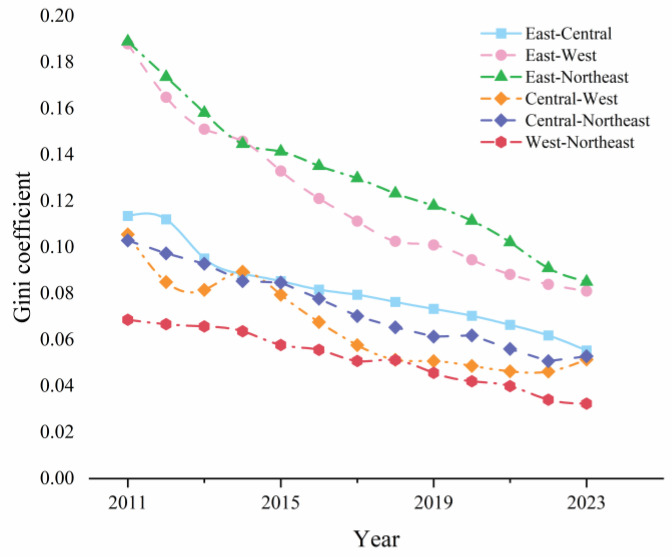
Inter-Regional Disparities of CCD-URBE (2011–2023).

To fully reveal the characteristics of disparities in China’s CCD-URBE, after analyzing the overall disparities and intra-regional disparities, this study continued to use the Gini coefficient to measure disparities between different regions. This provides a quantitative basis for precise policy-making in different regions. Figure 6 reports the inter-regional disparities of CCD-URBE from 2011 to 2023. On the whole, the disparities of CCD-URBE among the six regional combinations all showed a continuous downward trend. However, there were significant differences in the decline stage, speed and extent. This study classifies these trends into three types: rapid narrowing type, gentle narrowing type, and stable-then-narrowing type.

1. Rapid Narrowing Type. The specific regional combinations are East–West and East–Northeast. These two regional combinations had the largest decline range and fastest speed in Gini coefficient. They were the groups with the most significant improvement in inter-regional disparities. The decline rates reached 56.86% and 55.0%, respectively, with an average annual decline rate of 4.37% and 4.23%. Specifically, the East–West combination can be subdivided into two stages: “rapid decline” and “gentle decline”. From 2011 to 2017, the special investment for rural basic education in China’s “Western Development Strategy” was fully implemented. The Gini coefficient decreased from 0.188 to 0.111, a 40.96% decline in 6 years. From 2018 to 2023, the development of rural basic education in the western region entered a quality improvement period. The Gini coefficient decreased from 0.103 to 0.081, a 21.36% decline in 5 years. The speed of narrowing the disparity with the eastern region slowed down. The East–Northeast combination showed the characteristic of “uniform decline”. During the study period, the disparity kept decreasing at a constant speed. It dropped from 0.189 in 2011 to 0.085 in 2023, with no obvious stage differentiation.

2. Gentle Narrowing Type. The specific regional combinations are East–Central and Central–Northeast. These two regional combinations had a relatively low initial Gini coefficient. Their decline range was relatively gentle, and the disparity narrowing was mainly based on steady optimization. Specifically, the Gini coefficient of the East–Central combination decreased from 0.114 to 0.055, a decline of 51.75%, with an average annual decline rate of 3.98%. The synergy of urban–rural basic education development between the eastern and central regions continued to strengthen. The Gini coefficient of the Central–Northeast combination decreased from 0.103 to 0.0523, a decline of 49.22%, with an average annual decline rate of 3.79%. It showed a trend of “fast first, then stable”.

3. Stable-Then-Narrowing Type. The specific regional combinations are Central–West and West–Northeast. The early disparities of these two regional combinations were relatively stable. In the later period, due to differences in growth rates, the disparity of CCD-URBE entered a stage of rapid narrowing. Specifically, the Gini coefficient of the Central–West combination decreased from 0.106 to 0.052, a decline of 50.94%, with an average annual decline rate of 3.92%. It can be divided into two stages: stable fluctuation and rapid narrowing. From 2011 to 2018, the growth rates of rural basic education in the two regions were similar, and the Gini coefficient showed no obvious downward trend. After 2019, the growth rate of CCD-URBE in the western region further increased, while the growth rate in the central region tended to be gentle. The disparity between the two narrowed rapidly. The Gini coefficient of the West–Northeast combination decreased from 0.069 to 0.032, a decline of 53.62%, with an average annual decline rate of 4.12%. It showed the characteristic of “stable in the early stage and accelerated in the later stage”. From 2011 to 2017, the growth rates of the two regions were basically the same, and the Gini coefficient fluctuated slightly. After 2018, the disparity in CCD-URBE between the two narrowed at an accelerated rate. It became the combination with the smallest disparity at the end of the study period among the six regional combinations.

### 4.4. Sources and Contributions of Regional Disparities

Table 4 reports the contribution rate of regional disparities in CCD-URBE. It can be seen that during the study period, the contribution rate of inter-regional disparities remained at the highest level. Its average value reached 65.26%, with an annual fluctuation range concentrated between 63.16% and 68.84%. Disparities in CCD-URBE among the four major regions are the most important factor contributing to the overall disparity. This may be because the eastern region, relying on its strong financial capacity, has significantly higher investment in rural basic education than other regions. However, the central, western and northeastern regions are limited by their economic foundations. Their supply capacity of rural basic education resources is relatively weak. The disparity in CCD-URBE between regions persisted throughout the study period.

The fluctuation range of intra-regional disparity contribution rate was 21.85–23.69%, which was the most stable. This indicates that the disparities in CCD-URBE among provinces within the same region have a relatively limited impact on the overall disparity.

The contribution rate of transvariation density increased from 9.31% to 13.58%, with an increase of 45.87%. This was the largest increase among the three contribution items. This shows that the regional overlap effect of CCD-URBE is constantly strengthening. More and more provinces in the central, western and northeastern regions have higher CCD-URBE than some individual provinces in the eastern region. Their impact on the overall disparity is gradually expanding. In general, China’s balanced development policies have achieved remarkable results. They have continuously promoted the narrowing of inter-regional disparities during the study period. In the future, more attention should be paid to intra-regional refined regulation and positive guidance of transvariation density.

### 4.5. Markov Chain

To further analyze the spatiotemporal evolution characteristics of China’s CCD-URBE, this study used the quartile method to discretize the coupling coordination level into four states: low level (1), medium-low level (2), medium-high level (3), and high level (4). On this basis, a traditional Markov transition probability matrix and a spatial distribution-based Markov transition probability matrix were constructed [51,52]. Among them, the transition from a low level to a high level is defined as an upward transition, and the transition from a high level to a low level is defined as a downward transition. Table 5 reports the traditional Markov transition probability matrix of CCD-URBE types in China during the study period. Based on the data in the table, the following findings can be drawn:

First, the diagonal transition probability is dominant, and the CCD-URBE has strong stability. All diagonal elements in the matrix are significantly higher than non-diagonal elements, and the diagonal probabilities all exceed 70%. The probabilities of maintaining stability for regions with low-level, medium-low-level, and medium-high-level CCD-URBE are 74.26%, 71.29%, and 79.78% respectively. China’s CCD-URBE has a strong path dependence. Most regions tend to maintain their original coupling coordination grade during the study period. Fundamental changes are difficult to occur in the short term, and the stability characteristic is significant. Meanwhile, the CCD-URBE shows an obvious “club convergence” characteristic. On the one hand, the probability of high-level regions maintaining stability is 100%, and there are no cases of downward transition. On the other hand, the probability of low-level regions maintaining stability reaches 74.26%, and they cannot cross to medium-high or high levels. Although medium-low and medium-high level regions have the possibility of upward transition, their stability probability still exceeds 70%, showing a certain degree of club stickiness.

Second, the probability of “leapfrog” development of CCD-URBE types between adjacent years is small. The transition probabilities of non-adjacent types in the matrix are all 0. This indicates that the transition of CCD-URBE is subject to rigid constraints of adjacent progression. The narrowing of the gap in CCD-URBE is a gradual process. It is restricted by factors such as the allocation of educational resources, regional economic foundation, and policy implementation cycle. Breakthrough improvements are difficult to achieve in the short term.

Considering the interaction between spatially adjacent regions, this study added spatial lag terms to the traditional Markov chain model to construct a spatial Markov transition probability matrix. It compared and analyzed the transition probability of CCD-URBE under different neighborhoods, and explored the potential impact of the neighborhoods [51,52]. Table 6 reports the results of the spatial Markov chain. According to the results, the following findings can be drawn:

First, neighborhood type significantly changes the transition probability, and the economic and geographical conditions play a key role. By comparing the traditional matrix and the spatial matrix, it can be found that the coupling coordination type of the neighborhood has a significant moderating effect on the transition probability. For low-level regions, the probability of upward transition to a medium-low level in the traditional matrix is 25.74%. However, in the spatial matrix, when the neighborhood is at a low level (1), this transition probability decreases to 16.18%. For medium-low-level regions, the probability of upward transition to medium-high level is 12.50% when the neighborhood is at a low level (1). It rises to 33.33% when the neighborhood is at a medium-high level (3). This fully indicates that the higher the CCD-URBE of the neighborhood, the more positive spillovers the local region receives, and the stronger the motivation for upward transition.

Second, the local CCD-URBE type has a high degree of synergy with the neighborhood type. When the neighborhood is at a low level (1), the number of local low-level samples is significantly greater than that of other types. When the neighborhood is at a high level (4), the number of local high-level samples is also larger. In addition, when the neighborhoods are at the medium-low level (2) or medium-high level (3), the number of local samples of the corresponding type is also more.

Third, the neighborhood type affects the transition direction. When adjacent to high-level neighborhoods, the probability of upward transition of the region increases significantly. When adjacent to low-level neighborhoods, the probability of upward transition of the region decreases obviously. For example, for the medium-high level regions, the probability of upward transition to high level is 23.08% when the neighborhood is at medium-low level (2). It rises to 27.27% when the neighborhood is at high level (4).

Fourth, the spatial spillover effect strengthens club convergence. When the neighborhood is low-level, the probability that the low-level region maintains stability (83.82%) is higher than that of the traditional matrix (74.26%), and the upward transition probability (16.18%) is lower than that of the traditional matrix (25.74%), thereby strengthening the low-level club. When the neighborhood is high-level, the probability that the medium-high level region transfers upward to the high level (27.27%) is higher than that of the traditional matrix (20.22%), promoting more regions to enter the high-level club. The spillover effect of neighborhoods makes provinces with different CCD-URBE types form cluster distribution in geographical space, which further consolidates the “club convergence” pattern.

## 5. Convergence of CCD-URBE

### 5.1. σ Convergence

**Figure 7:**
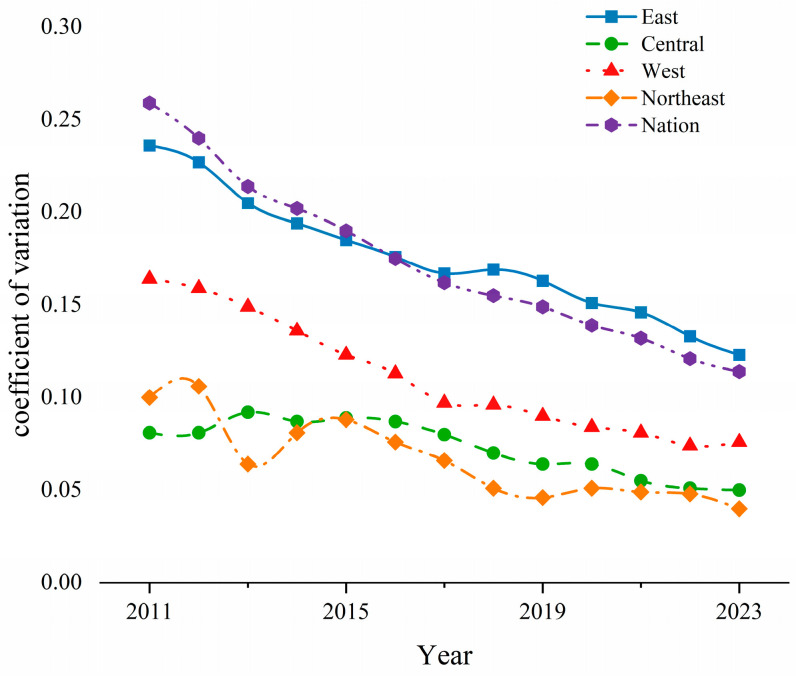
σ Convergence Evolution Trend of CCD-URBE at National and Regional Levels (2011–2023).

This study tested the σ convergence of CCD-URBE by measuring the coefficient of variation of CCD-URBE at the national level and in the four major regions during the study period [14,38]. The results are shown in Figure 7.

(1) From the perspective of evolution trend, the coefficient of variation of CCD-URBE showed a continuous downward trend at the national level. It showed a relatively obvious downward trend from 2011 to 2013, decreasing from 0.259 to 0.214. This may be attributed to “The National Medium and Long-Term Plan for Education Reform and Development (2010–2020)” launched by China during this period. The plan increased support for rural basic education in the early stage, and various measures began to show results. It continued to maintain a slow downward trend from 2013 to 2023. The coefficient of variation at the end of the study period was 0.114, with an average annual decline rate of about 4.3% and a total decline of 56%. The gap in national urban–rural basic education was constantly narrowing, and the CCD-URBE was gradually improving, showing a significant σ convergence pattern.

(2) From a regional perspective, the coefficient of variation of CCD-URBE in the four major regions generally showed a fluctuating downward trend. The decrease in magnitude and speed was considerable. Therefore, the CCD-URBE in the four major regions generally showed a σ-convergent pattern during the observation period.

In summary, the CCD-URBE at the national level and in the four major regions all showed σ convergence patterns during the study period. The gap in CCD-URBE generally showed a narrowing trend, which was basically consistent with the analysis results of the Gini coefficient mentioned earlier.

### 5.2. β Convergence

#### 5.2.1. Spatial Correlation Test

This study used the Moran’s I to examine the spatial correlation of CCD-URBE. The Global Moran’s I is a commonly used indicator to test whether a variable has spatial autocorrelation, with a value range of [−1, 1]. A value greater than 0 indicates positive spatial autocorrelation—the closer the value is to 1, the stronger the positive correlation. Conversely, a value less than 0 indicates negative spatial autocorrelation—the closer the value is to −1, the stronger the negative correlation. A value of 0 means there is no spatial autocorrelation between variables [54,55]. According to the results reported in Table 7, the Moran’s I of CCD-URBE in each year is greater than 0 and statistically significant at the 1% level. This indicates that there is a significant positive spatial autocorrelation in CCD-URBE during the study period.

#### 5.2.2. Absolute β Convergence

For the absolute β convergence test, it is necessary to first determine whether there is a spatial effect through the spatial correlation test, and then select a fixed-effect model or random-effect model based on the Hausman test [53]. Table 8 reports the test results. The results show the following:

First, model selection based on spatial correlation test and Hausman test. ① National level: The spatial lag LM = 19.963 ***, spatial error LM = 38.879 ***, Robust LM-error = 19.354 ***, and Robust LM-lag = 0.438. There exists significant spatial autocorrelation, with the dominant effect being the spatial error effect. The Hausman test result (75.99 ***) is significant, so the two-way fixed-effect model is adopted. Both the Wald and LR tests are significant, rejecting the simplification assumption of the SDM (Spatial Durbin Model). Finally, the two-way fixed-effect SDM is selected. ② Eastern region: The spatial lag LM = 5.217 **, spatial error LM = 17.33, and Robust LM-lag = 4.902 **. There exists significant spatial autocorrelation, with the dominant effect being the spatial lag effect. The Hausman test result (0.01) is not significant, so the random-effect model is adopted. The Wald/LR tests indicate that the SDM can be simplified to the SAR (Spatial Autoregressive Model). Finally, the random-effect SAR is selected. ③ Central region: The spatial lag LM = 0.854, spatial error LM = 2.07, and neither Robust LM is significant. There is no significant spatial autocorrelation. The Hausman test result (11.26 ***) is significant, so the two-way fixed-effect model is selected. Finally, the two-way fixed-effect model is adopted. ④ Western region: The spatial lag LM = 0.008, spatial error LM = 0.888, and neither Robust LM is significant. There is no significant spatial autocorrelation. The Hausman test result (12.52 ***) is significant, so the two-way fixed-effect model is adopted. Finally, the two-way fixed-effect model is selected. ⑤ Northeastern region: The spatial lag LM = 1.417, spatial error LM = 1.237, and neither Robust LM is significant. There is no significant spatial autocorrelation. The Hausman test result (0.11) is not significant, so the random-effect model is adopted. Finally, the random-effect model is selected.

Second, the CCD-URBE at the national level and the four major regions exhibits significant absolute β convergence. The β values for the national level, eastern, central, western, and northeastern regions are −0.247, −0.112, −0.523, −0.219, and −0.102 respectively. This indicates that the growth rate of CCD-URBE is significantly negatively correlated with its initial level—that is, regions with lower initial CCD-URBE have faster subsequent growth rates. The central region has the largest absolute value of β, showing the strongest convergence trend. The national level and the western region have a medium β, indicating a stable convergence trend. The eastern and northeastern regions have the smallest absolute values of coefficient β, reflecting a weaker convergence trend.

Third, significant regional differences in convergence speed. The convergence speed of national CCD-URBE is 2.37%. This means that without considering other exogenous variables, the gap in CCD-URBE among provinces across the country narrows at a rate of 2.37% per year. Among the four major regions, the central region has the highest convergence speed, reaching 6.17%—far higher than other regions. This may be related to the central region’s recent policies such as coordinating the allocation of urban–rural educational resources and improving rural school-running conditions. Provinces with lower initial CCD-URBE have achieved rapid catch-up, significantly narrowing the internal gap within the region. The western, eastern, and northeastern regions follow, with convergence speeds of 2.06%, 0.99%, and 0.90% respectively.

In summary, both the national level and the four major regions exhibit significant absolute β-convergence in terms of CCD-URBE. Moreover, the central region with low CCD-URBE has a higher convergence speed.

#### 5.2.3. Conditional β Convergence

This section further investigates whether CCD-URBE exhibits conditional β-convergence when four control variables are taken into account: urban–rural per capita income ratio (URI), per capita book collection (CLH), per capita GDP (GDP), and urbanization rate (URR). Among these variables, the per capita GDP has been logarithmically transformed (Ln (GDP)). Table 9 reports the results of conditional β convergence test for the CCD-URBE.

First, model selection based on spatial correlation test and Hausman test: Following the same procedure as in the previous subsection, based on the test results, the selected models are as follows: ① National level: the two-way fixed-effect SAR. ② Eastern region: the two-way fixed-effect SDM. ③ Central region: the two-way fixed-effect SEM. ④ Western region: the two-way fixed-effect model. ⑤ Northeastern region: the random-effect model.

Second, significant conditional β convergence exists in all regions. The national level and the four major regions (eastern, central, western, and northeastern) all show significant conditional β convergence. The β coefficients are −0.261, −0.305, −0.572, −0.233, and −0.285 respectively. Compared with absolute β convergence test, the absolute values of β coefficients at the national level and in the four major regions have increased. This indicates that after controlling the selected variables, the growth rate of CCD-URBE remains significantly negatively correlated with its initial level. In other words, regions with lower initial CCD-URBE have faster growth rates after excluding the interference of exogenous variables. After excluding the interference of control variables on the explained variable, the convergence speeds of the regions are in the order of central region (7.07%), eastern region (3.03%), northeastern region (2.80%), national level (2.52%), and western region (2.21%). Among them, the central region has the largest absolute value of β coefficient, far exceeding other regions, indicating that this region has the most sufficient convergence momentum.

Third, differential impacts of the four control variables. The impact of *URI* (urban–rural per capita income ratio) is significantly negative only at the national level and in the western region. This indicates that narrowing the urban–rural income gap can improve CCD-URBE in these two units—a smaller income gap means stronger rural household investment capacity in education and higher efficiency of government resource coordination. It is not significant in the eastern, central, and northeastern regions. This may be because the income gap in the eastern and central regions is already within a reasonable range, and its restrictive effect is relatively weak. In the northeastern region, the problem of population outflow is quite serious, and the narrowing of the income gap is difficult to have an impact on the education sector.

The impact of *CLH* (per capita book collection) is significant only in the eastern and central regions. It is significantly negative in the eastern region, possibly because cultural resources in the east are already saturated, leading to a decline in investment efficiency. It is significantly positive in the central region, which may be due to the still-shortage of cultural resources there—increased investment can enrich rural education content and narrow the urban–rural basic education gap. It is not significant at the national level, in the western region, or in the northeastern region, possibly because resource distribution is uneven and investment does not match demand in these regions, resulting in an unclear impact.

The impact of Ln(*GDP*) (per capita GDP) is significant only in the eastern and central regions. The coefficient in the eastern region is significantly positive, indicating that the economy and education are developing in a synchronized manner and the region is in a stage of high-quality development. The impact in other regions is not significant or even negative. This may be due to the fact that, except for the eastern region, the education finance in other regions has long relied heavily on central transfer payments. The economic growth has thus reduced the transfer payment intensity from the central government.

The impact of *URR* (urbanization rate) is significantly positive at the national level, in the eastern and central regions, indicating that in the process of urbanization, these regions pay attention to the matching of educational resources and the coordinated development of urban and rural basic education. In the West and Northeast, its impact is not significant, indicating that urbanization has not played a certain driving effect.

In summary, after controlling the variables such as economic development level, urban–rural income gap, social public resources, and urbanization to exclude exogenous interference, the CCD-URBE at the national level and in the four major regions all shows significant conditional β convergence, and the control variables have strengthened the convergence trend in most regions. At the same time, the impacts of the control variables show significant heterogeneity.

## 6. Conclusions and Policy Implications

### 6.1. Conclusions

Based on the data of 31 provinces in China during 2011–2023, this study measured the CCD-URBE using the entropy weight method, G1 order relation method, and coupling coordination degree model. On this basis, the Dagum Gini coefficient was adopted to decompose the disparities of CCD-URBE and identify the sources of these disparities. Traditional and spatial Markov chain models were used to study the evolution trend and reveal the development characteristics of CCD-URBE. Finally, the coefficient of variation and convergence test models were applied to examine the σ convergence, absolute β convergence, and conditional β convergence of CCD-URBE. The main research conclusions are as follows:

First, the overall imbalance of China’s CCD-URBE is low, and this imbalance shows a continuous narrowing trend. Policy inclination and regional resource radiation are the main factors driving this trend. In terms of intra-regional disparities, the internal disparities of the four major regions show significant stratification characteristics and all achieve continuous decline, but the process of narrowing disparities varies across regions. In terms of inter-regional disparities, the disparities among the six regional combinations also show a continuous downward trend. In terms of sources and contributions of regional disparities, inter-regional disparities are the most important source of the overall disparities, and the contribution rate of transvariation density to the overall disparities has the most significant increase.

Second, the results of traditional Markov chain model show that CCD-URBE has strong stability and path dependence characteristics. Most regions tend to maintain their original coupling coordination grade, and fundamental changes are difficult to occur in the short term. CCD-URBE shows the club convergence pattern and strong stickiness, and the probability of leapfrog transition is very small. Based on the spatial Markov chain model, it is found that the economic and geographical conditions of the neighborhood significantly moderate the transition probability. The higher the CCD-URBE of the neighborhood, the more positive spillovers the region obtains, and the greater the probability of upward transition. The CCD-URBE of the region and the neighborhood has strong synergy. The level of the neighborhood moderates the transition direction. The high-level neighborhood promotes the local upward, whereas the low-level neighborhood inhibits the local upward.

Third, the CCD-URBE of the nation and the four major regions shows obvious trends of σ-convergence, absolute β-convergence, and conditional β-convergence. The central region, which has a lower CCD-URBE, exhibits higher convergence speed. Meanwhile, the four control variables show significant heterogeneity in their impacts in different regions.

### 6.2. Policy Implications

Based on the above conclusions, the following policy implications can be drawn:

(1) Attach importance to the overall and regional disparity characteristics of CCD-URBE. The overall and regional disparities of CCD-URBE continue to narrow. However, it is worth noting that the intra-regional disparities show a hierarchical pattern of “the highest in the East, the second in the West, and the lower in the central and northeast”. The inter-regional disparities of the six major combinations are differentiated in stages. And inter-regional disparities are the main source of overall disparities. Therefore, for intra-regional disparities, it is necessary to guide the provinces with high CCD-URBE levels within the region to provide support to those with low CCD-URBE levels. For inter-regional disparities, the central government needs to provide a certain degree of financial inclination to underdeveloped regions and improve their school-running resources for rural basic education.

(2) Attach importance to the Markov transition probability and club convergence characteristics of CCD-URBE. CCD-URBE exhibits strong path dependence and significant club convergence, and the neighborhood type moderates transition probability. Therefore, policy-making should focus on breaking through the current stickiness. For low-level clubs, pilot provinces can be selected to introduce education management models from the regions with medium or high-level CCD-URBE. They can also prioritize cross-regional educational cooperation projects. This can break existing hierarchical constraints through online education and cross-regional teacher exchanges. For high-level clubs, the focus should be on guiding them to leverage their radiating and leading roles. This can transform the positive spillover effects of high-level neighbors into an upward driving force for low-level regions.

(3) Attach importance to the convergence and spatial linkage characteristics of CCD-URBE. The national level and the four major regions show significant absolute β convergence and conditional β convergence, and the convergence speed in the central region is faster. Therefore, for the central region, it is necessary to continuously optimize the allocation of urban and rural education resources and consolidate its convergence momentum. For the West, where the convergence rate is slow, it may be necessary to focus on the low level of economic development. At the national level, reducing the income gap between urban and rural areas and vigorously developing urbanization can accelerate the convergence of CCD-URBE to a certain extent, and promote the CCD-URBE to a higher equilibrium stage.

### 6.3. Limitations and Prospects

This study has some limitations. On the one hand, regarding the selection of family support indicators, due to data availability, the study mainly included quantifiable indicators such as family education funding input and family attention to education, while excluding implicit factors like differences in family upbringing styles and parental educational concepts. Although the impact of these factors on the coordinated development of urban–rural basic education is difficult to measure accurately, they are still of certain importance. On the other hand, for the sake of model simplicity, the Markov chain model employed an economic–geographic weight matrix, excluding spatial correlations arising from cultural and policy factors. However, incorporating cultural and policy factors into the spatial weight matrix may enhance the explanatory power of the model results.

In response to the above limitations, future research can be gradually improved from two aspects. On the one hand, for indicators in the family dimension, combining with practical situations and building on existing research, supplementary micro survey data can be integrated. Information such as parental educational concepts and upbringing behaviors can be collected through questionnaires to achieve a comprehensive portrayal of family participation in education. On the other hand, future research on basic education could explore the construction of a more comprehensive spatial weight matrix that incorporates economic, geographic, cultural, and policy factors. This approach would not only enrich spatial statistical theory but also contribute to more reliable model results.

## Figures and Tables

**Table 1:** Evaluation Indicator System for the CCD-URBE.

First-Level Indicator	Second-Level Indicator	Code	Unit
Teacher Quality	Proportion of kindergarten principals and full-time teachers with bachelor’s degrees	A1	%
	Kindergarten teacher-to-student ratio	A2	Teachers/Student
	Proportion of full-time primary school teachers with bachelor’s degrees	A3	%
	Primary school teacher-to-student ratio	A4	Teachers/Student
	Proportion of full-time junior high school teachers with master’s degrees	A5	%
	Junior high school teacher-to-student ratio	A6	Teachers/Student
	Proportion of full-time senior high school teachers with master’s degrees	A7	%
	Senior high school teacher-to-student ratio	A8	Teachers/Student
School-running Conditions	Per capita green space area in kindergartens	B1	m^2^/Person
	Per capita sports field area in kindergartens	B2	m^2^/Person
	Per capita school building area in primary schools	B3	m^2^/Person
	Per capita number of books in primary schools	B4	Volumes/Person
	Per capita number of computers in primary schools	B5	Units/Person
	Per capita total fixed assets in primary schools	B6	Ten Thousand Yuan/Person
	Per capita classroom building area in junior high schools	B7	m^2^/Person
	Per capita library building area in junior high schools	B8	m^2^/Person
	Per capita sports field area in junior high schools	B9	m^2^/Person
	Per capita fixed assets in junior high schools	B10	Ten Thousand Yuan/Person
	Per capita laboratory building area in senior high schools	B11	m^2^/Person
	Per capita sports field area in senior high schools	B12	m^2^/Person
	Per capita number of books in senior high schools	B13	Volumes/Person
	Per capita fixed assets in senior high schools	B14	Ten Thousand Yuan/Person
Educational Funds	Per capita educational funds in primary schools	C1	Thousand Yuan/Person
	Per capita educational funds in junior high schools	C2	Thousand Yuan/Person
	Per capita educational funds in senior high schools	C3	Thousand Yuan/Person
Family Support	Per capita educational consumption expenditure	D1	Yuan/Person
	Per capita family attention to education	D2	Times/Person
Educational Quality	Average years of education received per person	E1	Year

**Table 2:** Classification Standard for Coupling Coordination Degree.

Type	Range	Sub-Type
Coordinated Development	0.7 ≤ E ≤ 1.0	High-quality Coordination
	0.6 ≤ E < 0.7	Good Coordination
Transitional Development	0.5 ≤ E < 0.6	Barely Coordination
	0.4 ≤ E < 0.5	Mild Imbalance
Imbalanced Decline	0.3 ≤ E < 0.4	Moderate Imbalance
	0 ≤ E < 0.3	Severe Imbalance

**Table 3:** Descriptive Statistics of Control Variables.

Variable Name	Variable	Mean	Standard Deviation	Minimum	Maximum	Observations
Urban–Rural Per Capita Income Ratio	*URI*	2.538	0.384	1.794	3.672	403
Per Capita Book Collection	*CLH*	0.771	0.541	0.190	3.399	403
Per Capita GDP	*GDP*	60,350.521	32,039.840	16,023.830	200,278.000	403
Urbanization Rate	*URR*	59.681	12.923	22.810	89.600	403

Note: The data are sourced from the statistical yearbooks of various provinces in China during the study period: China Urban–Rural Construction Statistical Yearbook, China Rural Statistical Yearbook, China National Economic and Social Development Statistical Bulletin, and the provincial annual statistical data from the National Bureau of Statistics of China.

**Table 4:** Contribution Rate of Regional Disparities.

Year	Intra-Regional		Inter-Regional		Transvariation Density	
	Disparity	Contribution Rate (%)	Disparity	Contribution Rate (%)	Disparity	Contribution Rate (%)
2011	0.029	21.850	0.090	68.840	0.012	9.310
2012	0.028	22.830	0.079	65.520	0.014	11.650
2013	0.026	23.050	0.071	64.120	0.014	12.840
2014	0.024	23.050	0.067	64.140	0.013	12.820
2015	0.022	22.920	0.064	65.580	0.011	11.500
2016	0.021	23.040	0.059	65.610	0.010	11.350
2017	0.019	22.840	0.056	66.830	0.009	10.340
2018	0.019	23.600	0.051	65.020	0.009	11.380
2019	0.018	23.210	0.050	65.940	0.008	10.860
2020	0.016	22.920	0.047	66.040	0.008	11.030
2021	0.016	23.690	0.043	64.300	0.008	12.020
2022	0.015	23.680	0.039	63.320	0.008	13.010
2023	0.014	23.260	0.037	63.160	0.008	13.580

**Table 5:** Traditional Markov Chain Transition Probability Matrix.

t/t + 1	1	2	3	4	*n*
1	0.7426	0.2574	0	0	101
2	0	0.7129	0.2871	0	101
3	0	0	0.7978	0.2022	89
4	0	0	0	1	81

**Table 6:** Spatial Markov Chain Transition Probability Matrix.

Field Type	t/t + 1	1	2	3	4	*n*
1	1	0.838	0.162	0	0	68
	2	0	0.875	0.125	0	8
	3	0	0	0	0	0
	4	0	0	0	1	3
2	1	0.563	0.438	0	0	32
	2	0	0.740	0.260	0	50
	3	0	0	0.770	0.231	13
	4	0	0	0	1	8
3	1	0	1	0	0	1
	2	0	0.667	0.333	0	42
	3	0	0	0.833	0.167	54
	4	0	0	0	1	28
4	1	0	0	0	0	0
	2	0	0	1	0	1
	3	0	0	0.727	0.273	22
	4	0	0	0	1	42

**Table 7:** Moran’s I of CCD-URBE in China (2011–2023).

Period	2011	2012	2013	2014	2015	2016	2017
Moran’s I Index	0.482	0.477	0.47	0.453	0.443	0.432	0.429
Z	4.818	4.747	4.649	4.487	4.414	4.312	4.307
P	0	0	0	0	0	0	0
period	2018	2019	2020	2021	2022	2023	
Moran’s I Index	0.412	0.432	0.431	0.41	0.412	0.405	
Z	4.146	4.346	4.296	4.117	4.081	3.995	
P	0	0	0	0	0	0	

**Table 8:** Absolute β Convergence of CCD-URBE.

	Nation	East	Central	West	Northeast
	FE-SDM	RE-SAR	FE	FE	RE
β	−0.247 ***	−0.112 ***	−0.523 ***	−0.219 ***	−0.102 **
	(−10.48)	(−7.81)	(−5.72)	(−6.31)	(−2.12)
_cons			−0.480 ***	−0.153 ***	−0.037
			(−4.67)	(−3.89)	(−0.83)
λ	−0.04	0.074			
	(−0.41)	−0.59			
Convergence rate	2.37%	0.99%	6.17%	2.06%	0.90%
Individual fixation	Yes	No	Yes	Yes	No
Time fixation	Yes	No	Yes	Yes	No
LM—spatial lag	19.963 ***	5.217 **	0.854	0.008	1.417
Robust LM—spatial lag	0.438	4.902 **	0.484	1.459	0.182
LM—spatial error	38.879 ***	17.330	2.070	0.888	1.237
Robust LM—spatial error	19.354 ***	17.015	1.700	2.340	0.001
Husman	75.990 ***	0.010	11.260 ***	12.520 ***	0.110
*N*	372	120	72	144	36
R2	0.583	0.504	0.712	0.709	0.708

Note: ** *p* < 0.05, *** *p* < 0.01.

**Table 9:** Conditional β Convergence of CCD-URBE.

	Nation	East	Central	West	Northeast
	FE-SAR	FE-SDM	FE-SEM	FE	RE
β	−0.261 ***	−0.305 ***	−0.572 ***	−0.233 ***	−0.285 *
	(−10.47)	(−6.32)	(−10.44)	(−5.53)	(−1.87)
*URI*	−0.042 **	−0.060	−0.032	−0.069 *	−0.015
	(−2.32)	(−1.40)	(−0.70)	(−1.88)	(−0.12)
*CLH*	−0.006	−0.053 ***	0.107 **	0.003	0.119
	(−0.51)	(−4.55)	(2.07)	(0.13)	(0.97)
Ln(*GDP*)	−0.001	0.076 **	−0.063 **	0.042	0.001
	(−0.04)	(2.06)	(−2.00)	(1.02)	(0.00)
*URR*	0.002 ***	0.002 **	0.027 ***	−0.001	−0.003
	(3.53)	(2.22)	(8.00)	(−0.23)	(−0.90)
_cons				−0.362	−0.076
				(−0.79)	(−0.05)
λ or ρ	−0.071	−0.277 **	−0.469 **		
	(−0.79)	(−2.33)	(−2.19)		
Convergence rate	2.52%	3.03%	7.07%	2.21%	2.80%
Individual fixation	Yes	Yes	Yes	Yes	No
Time fixation	Yes	Yes	Yes	Yes	No
LM–spatial lag	13.121 ***	3.849 *	7.523 ***	0.613	2.007
Robust LM–spatial lag	0.081	12.768 ***	2.758 *	0.029	0.435
LM–spatial error	20.552 ***	16.631 ***	4.997 **	0.719	1.577
Robust LM–spatial error	7.512 ***	25.550 ***	0.233	0.135	0.004
Husman	76.720 ***	2.260 ***	52.380 ***	16.260 ***	0.030
*N*	372	120	72	144	36
R2	0.339	0.162	0.14	0.763	0.710

Note: * *p* < 0.1, ** *p* < 0.05, *** *p* < 0.01.

## Data Availability

The raw data supporting the conclusions of this article will be made available by the authors on request.

## Abbreviations

The following abbreviations are used in this manuscript:

CCD-URBE	Coupled and coordinated development level of urban–rural basic education
CCD	The coupling coordination degree model
CV	The coefficient of variation
SDM	Spatial Durbin Model
SAR	Spatial Autoregressive Model
SEM	Spatial Error Model
URI	The urban–rural per capita income ratio
CLH	Per capita book collection
URR	The urbanization rate
GDP	Per capita GDP
$G_{w}$	The contribution of intra-regional disparities
$G_{nb}$	The contribution of inter-regional disparities
$G_{t}$	The contribution of transvariation density
